# Measuring efficiency of public hospitals under the impact of Covid-19: the case of Türkiye

**DOI:** 10.1186/s12962-023-00480-6

**Published:** 2023-09-25

**Authors:** Seher Nur Sülkü, Alper Mortaş, Aziz Küçük

**Affiliations:** 1 Department of Econometrics, Ankara Haci Bayram Veli University, Emniyet Mahallesi Muammer Bostanci Caddesi No: 4, 06500 Ankara, Türkiye; 2 Directorate General for Public Hospitals, Ministry of Health, Ankara, Türkiye

**Keywords:** COVID-19, Health policy, Hospital efficiency, Türkiye, Stochastic frontier analysis, D22, D24, I11, I180

## Abstract

The Covid-19 pandemic has had serious medical, administrative and financial effects on the health system and hospitals around the world. In Türkiye, compared to 2019 realizations, in 2020 and 2021 respectively there were 39% and 21% decrease in the number of outpatient services and 29% and 17% decline in total inpatient services of public hospitals. The main subject of this research is how the pandemic period affects the Turkish public hospitals’ efficiency. We have measured the technical efficiency of outpatient and inpatient care services of Turkish public hospitals using Stochastic Frontier Analysis (SFA). The dataset includes 563 hospitals for the years 2015 through 2021. Inputs of number of physicians, nurses and other medical staff, and number of beds and their interactions with each other are introduced to the SFA models of outputs of outpatient visits and inpatient discharges adjusted with case mix index are derived. Firstly, we found that the years associated with Covid-19 have a significant negative impact on the inpatient service efficiency. Training and Research and City Hospitals have low efficiency scores in outpatient services but high efficiency scores in inpatient services. In addition, the regions with high population rates have positive impact in outpatient efficiency and negative impact in inpatient efficiency. During the pandemic, city hospitals, have received large investments, gained a key role by increasing both the patient load and their efficiency. Future reforms can be guided by taking advantage of the efficiency differences of hospitals in different environmental factors.

## Introduction

Covid-19, a novel coronavirus, has promptly disseminated all around the world after first detection in Wuhan-China in December 2019 [1] and not yet come to end as of December, 1, 2022 there were 640,071,389 total confirmed cases and 6,617,605 total deaths worldwide [2]. As highly contagious Covid-19 were spreading vastly, even the strongest health systems in the world were struggling to deal with Covid-19 cases and providing routine of non-Covid-19 healthcare. Over 90% of the countries reported disruptions in vital health services during 2020 [3]. Consequently, healthcare providers have been reorganized in order to tackle the impacts of pandemic, health authorities have called for reduction of hospital admissions except for urgent needs [4]. Many elective surgical procedures were postponed, the routine followed up and treatment of patients with chronic diseases were suspended, medical resources were redistributed, intensive care units, ventilators, healthcare personnel of other services were reassigned to Covid-19 patients during the pandemic [5, 6]. Indeed, telemedicine applications served to support outpatient services and manage healthcare organizations were useful to delivery of healthcare but also were responsible of for further reductions in service access [7, 8]. Moreover, the fear of infection of Covid-19, socio-economic barriers set by the pandemic, like curfews, stay at home orders, loss of jobs or income, were decreasing the demand and rising the unmet needs [8–10].

These factors caused huge reductions in policlinic visits, emergency department visits, inpatient admissions and hospitalization rate in all over the world [11–13]. For instance, there were more than a 40% decrease in emergency department patient volume in the USA [13]. American Hospital Association reports on average declines of 19.5% in inpatient volume and 34.5% in outpatient volume during 2020 relative to 2019’s baseline levels [14]. In Portugal, there were 48% reductions in emergency department visits in March 2020 and 57% in all hospitalizations from March to May 2020 [15, 16], and in Croatia 21% decrease in the total number of admissions [17]. These significant reductions in hospital services volumes caused severe decrease in revenues that could even affect hospitals’ and health systems’ abilities to serve their communities [14]. Moreover, the increased demand and broken supply chain led shortages for many drugs and hiked the prices of critical pharmaceuticals and thus further increased overall costs for hospitals [18].

Although Covid-19 began as a health crisis, pandemic worsen the preexisting economic fragilities both for advanced and emerging economies and become a primary economic crisis for many countries [19]. This situation restricted relief funds for hospitals and health systems. According to Devex’s funding platform as of June 27, 2020 the global funding dedicated to combating the Covid-19 exceeded $21.7 trillion, but only 1.3% were clearly related to health objectives while 0.8% were for vaccines and treatment, and 86% were to mitigate the economic impacts of the crisis [2]. As pandemic changed hospital’s organization, healthcare resource allocation both changes in the delivery of the healthcare along with demand sided changes and induced financial stress would have medium and long run effects on healthcare systems [5]. Therefore, evaluating the impact of the Covid-19 on hospitals efficiency is vital in order to reveal the pandemic’s effect on hospital sector and shed light to policy makers and hospital managers to employ wise strategies to deal with the inefficiencies and ensure the effective production and supply of healthcare [20]. At this point, our study contributes to literature by examining the technical efficiency of outpatient and inpatient care of public hospitals in Türkiye via SFA during the 2015–2021 period. The input–output data set of 563 public hospitals was gathered form the Ministry of Health (MoH) of Türkiye. We aim to evaluate the distribution of hospital efficiency scores by years, hospital types and socio-economic factors. Consequently, we compare the hospitals’ performances during the Covid-19 pandemic years to the pre-pandemic period trend.

In literature, there is no study examining impact of the pandemic on Turkish public hospitals efficiencies. Indeed, studies regarding Covid-19 impact on hospitals efficiency at micro level is less than a fingers of hand: Kamel and Mousa [20] for Egypt, Nepomuceno et al. [21] for Brazil, Caunic et al. [22] for Romania, and Henriques and Gouveia [23] for Portugal. Most of concurrent studies investigate country wise or state wise efficiencies of the health systems under Covid-19 at macro level such as: Kaye et al. [5], Xu et al. [24], Klumpp et al. [25], Breitenbach et al. [26]. Thus, our study offers a benchmark for future research and makes important contribution to literature examining Turkish public hospitals experience at micro level. In literature, prior to Covid-19 pandemic, there was a vast number of studies examining hospital efficiency commonly employing SFA and/or data envelopment approach as well documented [7, 27, 28]. Both methodologies have their advantages and disadvantages [29]. In this study, we prefer to apply SFA, as this econometric model introduces a term representing noise, measurement error, and exogenous shocks beyond the control of the production unit, this would be more convenient to study of unexpected Covid-19 pandemic’s effects on hospitals.

The rest of our study was organized as follows: first, Turkish hospital sector dynamics under the pressure of Covid-19 pandemic and Turkish health sector reforms for the 2000–2021 period has been discussed. Second, the SFA methodology is established and the data set has been introduced via basic descriptive analysis. Third, the findings obtained from SFA have been presented in the empirical findings section. Thereafter assessment of the findings has been discussed and the study is concluded.

### Turkish hospital sector under Covid-19 pandemic

In Türkiye, the first Covid-19 case was seen on March 11, 2020, there have been 16,919,638 total confirmed cases and 101,203 total deaths as of December, 1, 2022 [2]. In Türkiye, decisions to control the pandemic were given by central authority under the recommendations of scientific committees and WHO’s global guidance with the preliminary preparation of the Ministry of Health and the will of the Presidential Cabinet [30, 31]. After outbreak of pandemic healthcare delivery practices started to change with the increase in the demand for the care of the Covid-19. During the March–May 2020 period, the spread of diagnostic laboratories, early diagnosis and treatment, contact tracing and the management of medication and protective materials took place [31]. 794 hospitals, among a total of 1524 hospitals, were assigned as pandemic hospitals and 11,269 hospital beds were designated as isolation beds and field hospitals were established at the border gates [30]. Elective surgical procedures have been postponed to curb the spread of the disease and prioritize Covid-19 care. Healthcare via telemedicine have gained a place in daily practice to reduce the admission of patients [6, 32]. Moreover, pandemic control strategies like curfews, 'stay at home' campaigns and fear of infection have reduced hospital admissions. Consequently, the decrease in patient volume had negative effects on the income-expenditure balance of hospitals [33]. While outpatient and inpatient revenues of public hospitals decreased, their total expenses increased. The Turkish government has supported public hospitals through the global budget in 2021–2022. Social security institution contributed to the financial sustainability of all hospitals by providing intensive care and pandemic care reimbursement support. In Türkiye, total health expenditure increased by 41.6% in 2021 compared to the previous year and reached 353 billion 941 million TRY [34]. A phased reopening of the country taken place from May to June. In June, 2020 most of the Covid-19 related restrictions have been lifted in Türkiye including the resumption of healthcare [30, 35]. Since June 2020, most of the hospitals’ routine outpatient services and surgeries have been taking place. Moreover, Türkiye's Covid-19 vaccination program began in January, 2021, besides primary health care organizations, public and private hospitals offered vaccination service. Hence, the program was successfully implemented as more than 72% of the adult population have received their first dose [36]. At the beginning of July 2021, the MoH had been offering a third booster dose to the risk groups [37].

Though Türkiye has been considered as one of the most disciplined countries in fighting against Covid-19 [38, 39], hospital sector’s country-wise performance during the pandemic has not been examined yet, only some case reports on specific healthcare facilities exist such as Güleşen et al. [32]. Public hospitals have a very important place in the delivery of health care in Türkiye. In 2021, beds in public hospitals constituted 62% of the total number of hospital beds. In terms of health manpower, 58% of total doctors, 67% of nurses and 58% of health personnel are employed in public hospitals. When we look at the number of medical devices, 35% of the number of MRI devices, 55% of the number of CT and 60% of the number of Doppler USG are in public hospitals [40]. In this context, a better understanding of the technical efficiency of public hospitals is important in shaping national health policy.

### Turkish health sector reforms: 2000–2021

In Türkiye, Health Transformation Program (HTP) was initiated by the MoH in 2003. Since 2003, HTP has contributed to health access, improved health service delivery, better financial protection, and increased patient satisfaction. Within the framework of the autonomous public enterprises’ strategy gathered under a single roof, social security institution’s hospitals and hospitals affiliated to other public institutions and organizations were transferred to the MoH. The performance-based supplementary payment was put into practice in order to ensure that the restructured health organization is embraced by health personnel, especially physicians, and to increase their motivations. Since Jan, 2012 the Universal Health Insurance Law has become fully effective and general health insurance system has increased the population coverage from 80 to 87% [41]. Consequently, the number of physician visits per capita more than doubled, from 3.1 annually in 2002 to 8.0 in 2021. Infant mortality rates fell by almost a third, from 26 to 9.1 per 1000 births, and life expectancy grew to 78.6 [42].

Public hospital unions (PHUs) were established in 2012, and the public hospitals affiliated were reorganized as autonomous under the umbrella of PHUs. However, since PHUs were not well managed and multi-headedness emerged [43], they were abolished by a decree law in 2017, and hospitals were reassigned to provincial health directorates. Other regulations implemented within the scope of HTP were the "integrated health campuses" and "city hospitals" projects based on the public–private partnership model. While the financing of these projects, physical equipment, maintenance, repair and renewal works are provided by the private sector; health service delivery and customer guarantee in the project contract are provided by the state. It is a matter of interest how the city hospitals, which replaced the old established hospitals, changed the efficiency of the health system. Between 2017 and 2021, 13 city hospitals were opened and the construction process of 5 city hospitals have been continuing.

## Methods

### Stochastic frontier analysis

The stochastic frontier production models, first presented in the Cobb–Douglas form by Aigner et al. [44] and Meeusen and Broeck [45], are very well known and widely applied technique in the literature of hospital efficiency. In our study, by using the Turkish public hospitals data for the period of (2015, 2021), we apply the translog generation function, which is a generalization of the Cobb–Douglas function and is a flexible functional form that provides a quadratic approximation as follows:

1 $${\mathrm{lny}}_{\mathrm{it}}={\upbeta }_{0}+\sum_{\mathrm{j}=1}^{\mathrm{h}}{\upbeta }_{\mathrm{j}}{\mathrm{lnx}}_{\mathrm{jit}}+\frac{1}{2}\sum_{\mathrm{j}=1}^{\mathrm{h}}\sum_{\mathrm{k}=1}^{\mathrm{h}}{\upbeta }_{\mathrm{jk}}{\mathrm{lnx}}_{\mathrm{jit}}{\mathrm{lnx}}_{\mathrm{kit}}+\sum_{\uptau =2016}^{2021}{\upphi }_{\uptau }{\mathrm{D}}_{\uptau }+({\mathrm{v}}_{\mathrm{it}}- {\mathrm{u}}_{\mathrm{it}})$$

where $\mathrm{i}=1,\dots .,\mathrm{n}$, is the index for the hospital identity and both $\mathrm{j}=1,\dots ,\mathrm{h}$, and $\mathrm{k}=1,\dots ,\mathrm{h}$ for the input types, t = 2015, …, 2021 for the year. In Eq. (1) ${\mathrm{y}}_{\mathrm{it}}$ is ith hospital’s output production in time period t, ${\mathrm{x}}_{\mathrm{jit}}$ is jth type input amount of the ith hospital in time period t, ${\mathrm{D}}_{\uptau }$ is dummy variable based on year such as it takes 1 if t = τ and zero otherwise here and τ = 2016, …, 2021 for the year effects with 2015 providing the reference, ${\mathrm{u}}_{\mathrm{it}}$ is an inefficiency component with half normal distribution such as ${\mathrm{u}}_{\mathrm{i}}\sim {\mathrm{N}}^{+}(0,{\upsigma }_{\mathrm{u}}^{2})$ and finally ${\mathrm{v}}_{\mathrm{it}}$ is a random error term coming from ${\mathrm{v}}_{\mathrm{i}}\sim \mathrm{ N }(0,{\upsigma }_{\mathrm{v}}^{2})$. In this model, ${\mathrm{u}}_{\mathrm{it}}$ inefficiency term indicates the quantity that is less than the maximum feasible production frontier, while the ${\mathrm{v}}_{\mathrm{it}}$ term captures random variation between units across time. ${\mathrm{v}}_{\mathrm{it}}$ terms may result from measurement error or omitted factors [46]. It is assumed that the composite error term $({\mathrm{v}}_{\mathrm{it}}- {\mathrm{u}}_{\mathrm{it}})$ is skewed to the left [47]. In the model, ${\mathrm{lnx}}_{\mathrm{jit}}{\mathrm{lnx}}_{\mathrm{kit}}$ term, is the quadratic term when j = k and cross product when j ≠ k, helps to gain more degrees of freedom [27]. Also, Chirikos and Sear [48] states that cross products included in the translog function increase the average efficiency scores due to the increased flexibility of the function.

In the SFA, the problem of heteroscedasticity in error components introduced in Eq. (1) is used to address the extrinsic determinants of inefficiency [49, 50]. In Eq. (2) inefficiency effects model for panel data is defined as.

2 $${\mathrm{u}}_{\mathrm{it}}={\updelta }_{\mathrm{m}} {\mathrm{z}}_{\mathrm{mi}}+{\mathrm{w}}_{\mathrm{it}},$$

where ${\mathrm{z}}_{\mathrm{mi}}$ is explanatory variables associated with inefficiency; ${\updelta }_{\mathrm{m}}$ is unknown parameters to be estimated; and ${\mathrm{w}}_{\mathrm{it}}$ are unobservable random variables assumed to be independently distributed, obtained by truncation of normal distribution with mean zero and unknown variance [51]. These ${\mathrm{z}}_{\mathrm{mi}}$ terms are also called as inefficiency factors (or external factors) as if their coefficients are positive (negative) which means that they correlate positively (negatively) with the inefficiency component (${\mathrm{u}}_{\mathrm{it}}$) in Eq. (2) and thus have negative (positive) effect on the efficiency of the hospitals in Eq. (1). These external factors, ${\mathrm{z}}_{\mathrm{mi}}$, on the production function are not under control of the hospital management such as: government intervention in the market in which the firm produces, type of ownership of the firm, age of the workforce, as well as unpredictable stochastic variables related to production risks such as natural events, luck, strike, accident [46].

In our study, we pooled the panel data and use it as cross-sectional data. Pooled data technique allows time-varying inefficiency such that the random error and inefficiency components are independently and identically distributed or they can be heteroscedastic [52]. Thus, temporal variation in inefficiency, that is not possible in panel data, can be recorded by introducing a time trend variable or time dummies [50].

In SFA, primarily the production frontier is estimated by using the maximum estimation method for the regression. Then deviations from the limit are estimated. Technical efficiency measures the ratio of the observed output amount of the ith hospital to its potential output relative to the relevant input amount. Output-oriented technical efficiency can be written as follows:

3 $${\mathrm{TE}}_{\mathrm{it}}=\mathrm{exp}\left(-{\mathrm{u}}_{\mathrm{it}}\right)$$

This measure of technical efficiency takes a value between zero and one, efficiency improves as ${\mathrm{TE}}_{\mathrm{it}}$ converges to 1.

The output elasticities of each of the input variables, ${\mathrm{x}}_{\mathrm{j}}$, at their mean are formulated in Eq. (4) as:

4 $${\mathrm{e}}_{\mathrm{j}}=\frac{\partial \mathrm{lny}}{\partial {\mathrm{lnx}}_{\mathrm{j}}}={\upbeta }_{\mathrm{j}}+\sum_{\mathrm{j}}{\upbeta }_{\mathrm{jk}}{\mathrm{lnx}}_{\mathrm{j}}$$

that shows the estimated response of the outputs against a unit change in the inputs, here ${\upbeta }_{\mathrm{j}}$ represents the direct effect of input ${\mathrm{x}}_{\mathrm{j}}$ on the production and $\sum_{\mathrm{j}}{\upbeta }_{\mathrm{jk}}{\mathrm{lnx}}_{\mathrm{j}}$ represents total indirect effect comes from the interaction of input ${\mathrm{x}}_{\mathrm{j}}$ with itself i.e. quadratic terms and with other input variables i.e. cross product terms. In our study, “returns to scale” (RTS) that refers to how well a hospital is producing its products is also calculated for outpatient and inpatient production of hospitals. There are three types of returns to scales: Constant when the output rises in exactly same proportion when all the inputs increase proportionately, increasing if the output rises more than proportionately, decreasing if the output rises less than proportionately [53]. In this study, returns to scales are calculated as the sum of the input elasticities as following the formulation given below:

5 $$\mathrm{RTS}=\sum_{\mathrm{j}}{\mathrm{e}}_{\mathrm{j}}.$$

Depending on whether this estimate is more than, equal to, or less than one, the RTS will either be increasing, constant, or decreasing, respectively.

In literature, SFA and/or data envelopment approach (DEA) are the most common methodologies applied to measure hospital efficiencies [27, 28]. The DEA is a nonparametric method that creates an envelopment surface over the data points so that all observed input–output combinations fall on or below the frontier of production possibilities; and the technical efficiency is measured relative to the highest observed performance that lie on the frontier [54]. Both SFA and DEA approaches have different strengths and weaknesses. First, in order to apply the SFA method, the firm's production function, i.e. its functional form must be known. This situation that the functional form cannot be determined exactly raises the risk. But, as DEA is a nonparametric method, there is no need for such an assumption in DEA. While DEA can be used in cases where there is more than one firm output, the SFA method does not allow more than one output in technical efficiency measurement [29]. On the other hand, the main advantage of SFA over DEA is that it can distinguish factors beyond the hospital's control, such as exogenous shocks and measurement error, and express it as random error. In case of missing and erroneous data, reliability of DEA results becomes questionable [55]. Another weakness of DEA is that it is not based on any economic theory containing hypotheses. As a result, the unexpected effects of Covid-19 on hospitals data indicate that SFA would be more appropriate. In our study, we aim to determine a functional form and to reveal the effects of this period with hypothesis tests.

### Data set

Our study includes 563 public hospitals serving in Türkiye during the period of 2015–2021. Universities and private hospitals are not included in the study. In addition, in order to ensure the homogeneity of the data, we exclude the branch hospitals and E group hospitals with less than 25 beds. As a result, 478 general hospitals, 13 City Hospitals and 72 Training and Research Hospitals exist in our sample which is denoted %63 of total public hospitals in Türkiye [56]. The data is obtained from the General Directorate of Public Hospitals of MoH. Table 1 introduces the variables and presents the descriptive statistics of pooled data set.

**Table 1:** Variables and descriptive statistics, pooled data, 2015–2021

Variable	Definition	Mean	SD	Min	Max
Outputs					
Outpatient	Outpatient	569,947.2	610,541.3	12,458	4,348,969
Inpatientx*CMI*	Inpatient adjusted by *CMI*	12,122.5	17,984.1	0.7	306,377.4
Inputs					
Physician	Physician	101.8	175.8	3	3219
Nurse	Nurse	173.2	221.5	10	2735
OtherMedStaff	Other medical staff	157.6	173.8	12	2011
Bed	Bed	225.9	296.3	25	4881
Year dummies from *D*_*2016*_ to *D*_*2021*_	Takes 1 if the year is the matched one like for *D*_2016_ = 1 if the year *t* = 2016 and so on				
Inefficiency variables					
TRH	Training and research status	0.11	0.31	0	1
CITY	City hospital status	0.01	0.12	0	1
POP	Population ratio (%)	0.03	0.05	0.00	0.19
RATE	Exchange rate	5.15	2.12	2.72	8.98

Covid-outbreak *D*_2020_ takes one if the year is 2020

Covid-pandemic *D*_2021_ takes one if the year is 2021

Total Number of Observations: 3679

Total Number of Hospital: 563 (13 City Hospital, 72 Training and Research Hospital)

In our data set, the outputs include total outpatient services, as summation of the emergency department and policlinic visits, and total number of inpatients adjusted by case-mix index (CMI), whereas the inputs include total number of physicians, nurses, and other medical staffs employed, and total number of beds within the hospital ready for use. In literature, patient activities are commonly measured by discharge outputs in the recent line of research. However, since the dramatic variations of resource consumptions between patients’ classification, adjustment by a hospital service complexity-based index has become widespread in hospital efficiency analyses [54]. Rosko [57] adjusted discharge with case mix index in order to reflect cost variations associated with case-mix complexity. In this context, Ferreira and Marques [56] concluded that CMI does not change efficiency scores and ranking of hospitals remarkably, but it generates efficiencies with higher consistency. In our study, CMI values used for inpatient services of each hospital were gathered from the MoH. The index is based on treatment resources spent in cases [59].^1^

In addition, in our analysis, the time dummy variables are introduced as input variables. They capture technical change based on year, represents a trend towards quality improvements that account for differences in the production structure. Quality improvement trend is defined for the increasing trend. This can be explained by the advancement of medical technology, evidence-based guidelines for care delivery, or health policy changes [60].

Moreover, beyond the input–output mechanism, healthcare facilities’ (in)efficiencies might depend on their inherent characteristics and inhabited environment. In our study, we introduced Training and Research Status, City Hospital Status, Population Ratio, Dollar Exchange Rate and the 2020/21 year dummies of as external inefficiency factors. First of all, the 2020 and 2021 year dummies respectively ${\mathrm{D}}_{2020}$ and ${\mathrm{D}}_{2021}$ are introduced in order to capture the Covid-19 outbreak and the pandemic’s impact on the hospitals’ production. As well as economy, health system and all aspects of human life have been jeopardized due to Covid-19 pandemic in Türkiye [35], therefore the pandemic has inevitable effects on inherent characteristics and inhabited environment of hospitals. Second of all, hospital type is accepted as a very important determinant of efficiency [61]. Training and research hospitals tend to present a high-quality service structure that provides specialized care, management, and resource organization compared to general public hospitals, this situation might have either positive or negative effects on efficiency. Moreover, as mentioned before, city hospitals projects, one of the latest reforms of the Health Transformation Program, have been implemented with the public private partnership (PPP) since 2017. Private sector management skills are expected to be used to improve the technical quality and productive efficiency of city hospitals, each of which are built with an average of 1500 beds. With the PPP model, it was aimed to provide access to private finance for capital investments and to overcome constraints on public sector budgets for capital expenditures and/or recurrent expenditures [62].

Furthermore, as commonly used as external factor, population density is significantly correlated with hospital efficiency scores and one of the most important drivers of higher Covid-19 infection rates [63, 64]. We introduced the population ratio (POP) which is calculated as the ratio of the current population in that province to the total population of Türkiye. The data obtained from the Turkish Statistical Institute has sensitivity according to years by the address-based registration system [65]. The population density directly affects the demand for the healthcare services as population grows the healthcare needs also rise [66]. Moreover, also the population density would be the good proxy to measure the development level of the provinces that the health facility locates. As we may incorporate the higher development level of the cities with higher GDP per capita income levels and better social economic conditions [67, 68].

Finally, we introduced USD Dollar/TRY exchange rate (RATE) as an important external variable. In Türkiye, since 2018, there has been a currency crisis which deepened with the Covid-19 outbreak [69]. Indeed, pharmaceutical and medical products supply heavily depends on imports, as Türkiye has been among the top 25 importers of pharmaceutical products by 2020 and expected to be 10th as of 2023 [70]. Turkish lira’s depreciation and loss in purchasing power made financing of healthcare costlier for the hospitals and for every agent in economy from households to social security institution.

**Fig. 1:**
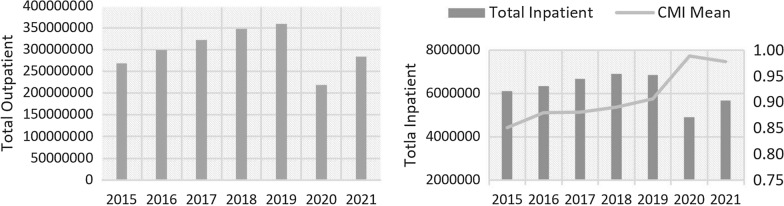
Total number of healthcare services and case-mix index (CMI): 2015–2021

As we have introduced our data set, now we briefly consider the trends in the hospital sector production under the effect of the Covid-19 pandemic. Total number of outpatients service as summation of policlinic and emergency department visits and total number of inpatient services along with the case mix index are given in Fig. 1 from 2015 to 2021.

As seen in the Fig. 1, the increasing trend in the total number of outpatient and inpatient services was broken in 2020. Although services provided for the Covid-19 cases were included in both accounts, there were dramatic decreases with the outbreak of the pandemic. Compared to 2019 realizations, in 2020 and 2021 respectively there were 39% and 21% decrease in the number of outpatient services and 29% and 17% decline in total inpatient services. The provided healthcare have not bounced back their pre-pandemic levels even after establishment of controlled normal life since June 2020 with removal of the most Covid-19 measurements and successfully implementation of Covid-19 vaccination program since January, 2021. On the other hand, mean of the case-mix index measuring the severity of the inpatient services sharply increased by 8.79% from 0.91 in 2019 to 0.99 level in 2020. It can be deduced that it is the result of giving priority to more severe cases as a policy of the MoH during the pandemic.

## Findings

Firstly, we applied Likelihood Ratio (LR) test to select the proper model to use for our data. The LR results presented in Table 2 indicate that the stochastic frontier estimation procedure is more appropriate than ordinary least square method. Then, the stochastic generation model described in methodology section was established separately considering the outpatient and inpatient care services as output (${\mathrm{y}}_{\mathrm{it}}$) in Eq. (1). The estimated parameters of frontier production function and inefficiency effects with *p*-values are presented in Table 2. For all calculations in this study, we benefited STATA 16 and specific source codes for SFA which is provided by Kumbhakar et al. [50].

**Table 2:** Estimation of parameters

	Outpatient	Inpatientx*CMI*
Constant	6.70 (0.000)***	− 0.08 (0.725)
Inputs $({x}_{j})$		
Physician	0.54 (0.000)***	0.08 (0.600)
Nurse	0.88 (0.000)***	0.76 (0.005)***
OtherMedStaff	0.61 (0.000)***	0.37 (0.109)
Bed		1.48 (0.000)***
Quadratic terms $({{x}_{j}}^{2})$		
Physicianxphysician	− 0.10 (0.043)**	− 0.26 (0.001)***
Nursexnurse	− 0.05 (0.653)	− 0.90 (0.001)***
OtherMedStaffxotherMedStaff	− 0.27 (0.033)**	− 0.22 (0.262)
Bedxbed		− 0.07 (0.649)
Cross products $$(x_j$$, $$x_k )$$		
Physicianxnurse	− 0.11 (0.071)*	0.39 (0.001)***
PhysicianxotherMedStaff	0.20 (0.001)***	− 0.01 (0.872)
NursexotherMedStaff	0.01 (0.913)	0.36 (0.043)**
Bedxphysician		− 0.13 (0.129)
Bedxnurse		0.14 (0.417)
BedxotherMedStaff		− 0.16 (0.214)
Year dummies		
*D*_2016_	0.05 (0.004)***	0.00 (0.908)
*D*_2017_	− 0.03 (0.134)	− 0.06 (0.026)**
*D*_2018_	− 0.02 (0.315)	− 0.10 (0.001)***
*D*_2019_	− 0.06 (0.017)**	− 0.15 (0.000)***
*D*_2020_	− 0.56 (0.000)***	− 0.49 (0.000)***
*D*_2011_	− 0.37 (0.000)***	− 0.35 (0.000)***
Inefficieny effects		
Constant	− 3.05 (0.000)***	− 1.02 (0.000)***
*TRH*	1.33 (0.000)***	− 1.73 (0.000)***
*CITY*	0.87 (0.001)***	− 1.86 (0.000)***
*POP*	− 6.43 (0.000)***	5.27 (0.000)***
*RATE*	0.00 (0.994)	0.04 (0.224)
*D*_2020_	0.21 (0.336)	0.25 (0.083)*
*D*_2021_	0.24 (0.441)	0.69 (0.001)***
Log Likelihood	− 734.15	− 2535.13
LR Test	238***	864***

*** Significant at the 0.01 level, ** significant at the 0.05 level, * significant at the 0.10 level

First, as explained in methodology section, the ${\upbeta }_{\mathrm{j}}$ coefficient of ${\mathrm{x}}_{\mathrm{j}}$ input variable indicates the direct effect of that input on the examined output. As seen in Table 2, all health personnel including physicians, nurses and other medical staffs have positive and statistically significant direct effects (with *p*-values < 0.01) on production of outpatients. For the production of inpatient services, even though each type of health personnel has positive direct effect, the contribution of nurses significantly stands out. Moreover, the bed size was considered as an input only for examining the inpatient services and its positive significant direct effect is measured as $\left( {{{{\hat{\beta }}}}_{{\text{Bed}}} = 1.48, p{\text{ - value}} < 0.01} \right)$.

The coefficients of quadratic terms show the responses of the input to the scale information. Accordingly, although their direct effects were positive, high increases in the volume of health personnel may have a negative effect on the number of services produced in hospitals. As seen in Table 2, all physicianxphysician, nursexnurse, and otherMedStaffxotherMedStaff quadratic terms have negative coefficients for both the outpatient and inpatient services production, thus the law of diminishing marginal productivity applies in Turkish public health sector. Indeed, these quadratic terms are significant at 5% level, except nursexnurse for the outpatient services and otherMedStaffxotherMedStaff for the inpatient services.

The cross-interaction coefficients indicate the complementarity or substitutability between the input variables. When we consider the outpatient services production, it is understood that 1% increase in the number of physicians reduces the need for nurses by − 0.11% (*p*-value = 0.071) and increases the need for other medical staff by 0.20% (*p*-value = 0.001). On the other side, they are physicians and nurses who are complementary of each other for the production of inpatient services as 1% rise in the physicians’ numbers requires 0.39% (*p*-value = 0.001) increase in the size of the nurses; and further nurses and other medical staff are also complementary of each other as 1% increase in nurses requires 0.36% (*p*-value = 0.043) increase in other staff. Furthermore, although not statistically significant, increase in bed size requires more nurses but not physicians.

In both productions of outpatient and inpatient services, the coefficients of the year dummy variables have negative trends. Compared to 2015, from 2017 to 2019 there were slight but increasing inefficiencies. However, this inefficiency trend became very severe with the hit of Covid-19 during the pandemic years of 2020 and 2021. Since negative year effect keep increasing, the models have created production limits that shift inward over the years.

Subsequently, we consider the effects of the inefficiency factors on hospital production. As explained in detail in the methodology section for SFA, when inefficiency factors are positively correlated with inefficiency component (${\mathrm{u}}_{\mathrm{it}}$) in Eq. (2), they decrease the efficiency of the hospitals in Eq. (1). Thus, as Training and Research Hospital (TRH) status is positive significant factor of inefficiency [with coefficient 1.33(*p*-value < 0.01)], it has negative effect on hospital outpatient services production efficiency, on contrast TRH has positive effect on inpatient services production efficiency. Furthermore, the type of hospital has significant effects on production efficiency. Firstly, being city hospital is negatively associated with the inefficiencies in inpatient services (− 1.86, *p*-value < 0.01), so it increases efficiency of production of inpatient services. On contrary, the situation is the opposite for outpatient services as being city hospital rises the inefficiencies.

Provincial population ratio (POP) creates different effects on outpatient and inpatient services. In provinces with a large population, the efficiency of outpatient services is high, but the efficiency of inpatient services is low. Additionally, exchange rate did not have any significant effect on either of the health services production process.

The dummy variables of years (${\text{D}}_{2020}$, ${\text{D}}_{2021}$) have been introduced to capture the pandemic effect on hospital’s inefficiencies. It was observed that, the year dummies do not have a significant impact on the efficiency of outpatient service. On the other hand, they tend to decrease inpatient service efficiency. The magnitude of the negative impact of 2021 is greater than that of 2020. Furthermore, when we classify the efficiency scores according to the years as in Table 4, we observed that the mean of scores decrease in pandemic years.

In Table 3, we measured the returns to scales (RTS) as following the formula given in Eq. (5) as the sum of the input elasticities, that were calculated following Eq. (4), for both outpatient and inpatient production. According to the output elasticities, the most important factor in outpatient service is the physicians with elasticity of 0.57 whereas the most important factor in inpatient service is the bed size with elasticity of 0.53. The sum of the outpatient elasticity coefficients is equal to 1.02, which shows that the production process has almost constant return to scale, that if we increase all inputs by 1%, the number of outpatient services increase by 1.02%. On the other hand, the inpatient production process has increasing returns to scale such that in the case all inputs rise by 1%, the number of inpatient services increase by 1.35%.

**Table 3:** Output elasticities of input variables

Inputs	Outpatient	Inpatientx*CMI*
Physician	0.57	0.18
Nurse	0.27	0.47
OtherMedStaff	0.18	0.17
Bed		0.53
Summation: *RTS*	1.02	1.35

Finally, in Table 4 the efficiency scores distributions of public hospitals are summarized regarding to outpatient and inpatient services.

**Table 4:** Efficiency scores

	Outpatient		Inpatientx*CMI*	
	Mean	SD	Mean	SD
All sample	0.78	0.11	0.63	0.19
Year				
2015	0.78	0.10	0.66	0.15
2016	0.78	0.11	0.66	0.17
2017	0.79	0.10	0.66	0.16
2018	0.79	0.11	0.66	0.17
2019	0.79	0.10	0.65	0.18
2020	0.76	0.11	0.61	0.20
2021	0.76	0.11	0.54	0.22
Hospital type				
*TRH*	0.68	0.14	0.76	0.09
City	0.72	0.15	0.80	0.10
Other	0.79	0.10	0.62	0.19
Regions				
Mediterranean	0.78	0.10	0.65	0.19
East Anatolia	0.76	0.11	0.62	0.19
Aegean	0.78	0.10	0.61	0.17
Southeast Anatolia	0.80	0.10	0.71	0.15
Central Anatolia	0.76	0.12	0.59	0.21
Black Sea	0.75	0.12	0.65	0.18
Marmara	0.81	0.09	0.64	0.17

Here *TRH* denotes Training and Research Hospitals

Actually, Table 4 allow us to double check our findings we obtained from the parameter estimations of the stochastic frontier models. Accordingly, for inpatient services similar score averages have been obtained for the years from 2015 to 2019 until 2020, where there has been a decrease in efficiency of health services due to the pandemic. It is a striking result that the inpatient efficiency in 2021 is lower than in 2020 despite the increase in the amount of output. When we made a detailed examination, there was a significant decrease in the inpatient efficiency of small-scale hospitals in 2021.^2^ For the outpatient services, we observe very slight decreasing effect of the Covid-19 on efficiency scores. Training and Research and City Hospitals have a low efficiency score in outpatient services and a high efficiency score in inpatient services. In this case, Training and Research Hospitals and City Hospitals focus on inpatient services with high case mix index levels. In the detailed analysis, we determine that the newly opened city hospitals have steadily increased their efficiency scores over the years. Moreover, when we look at the efficiency scores of geographical regions, we can deduce that regions with high population rates like Marmara have high scores in outpatient efficiency but low scores in inpatient efficiency.^3^

## Discussion

This study assesses the impact of the Covid-19 pandemic on the efficiency of the public MoH hospitals via SFA examining pre-pandemic and pandemic periods comparatively during the 2015–2021. Both outpatient and inpatient services production process are considered driving translog generation functions with input variables of total number of health personnel, hospital size and year dummies. Consequently, the elasticities of hospital production regarding to inputs are estimated, scales to returns are calculated and distribution of the technical efficiencies are obtained separately for outpatient and inpatient services.

First of all, it is observed that all health personnel including physicians, nurses and other medical staffs have positive and statistically significant direct effects on production of outpatients and inpatient services. Similar results were obtained in SFA efficiency analyzes conducted in hospitals in countries such as the USA, Germany, and Iran [71]. Indeed, contribution of nurses was prominent for the inpatient services. While examining the squared input variables, i.e. the quadratic terms, it is figured out that law of diminishing marginal productivity applies in Turkish public health sector. The physicians and the nurses are found as complementary of each other for the production of inpatient services. On the other hand, since the effect of nurses is also important in the production of outpatients, as Butler et al. [72] discussed, the substitution of nurses for doctors in some outpatient clinics like rheumatology clinics may be suggested. The replacement of physicians with nurses could help address physician shortages and lighten physician workload [73]. In addition, increase in bed size requires more nurses but not physicians. Besides, we revealed that inpatient services production was more productive than the outpatient services while the outpatient production has constant returns to scale, the inpatient production process has increasing returns to scale.

It can be seen that there were contractions in the limits of the stochastic frontier curves of production of out(in)patient services throughout years, that deepened severely with the hit of Covid-19 in 2020 and 2021. For the inpatient services the magnitude of the negative impact of 2021 is greater than that of 2020. Likewise, it can be seen that for inpatient services there was a decrease in efficiency of healthcare due to the pandemic in 2020 and 2021. Indeed, it is remarkable to see that although the amount of production was increased, the inpatient efficiency in 2021 was even lower than in 2020. Similarly, the outpatient services efficiency of hospitals decreased with the hit of Covid-19. As a result of the decrease in visits, especially in 2021, when doctors started to examine through the Central Physician Appointment System, outpatient efficiency in hospitals decreased. As of mid-2020, designated “pandemic hospitals” were usually TRH and city hospitals. In addition, while more outpatients were cared for, especially in small-scale hospitals, the number of inpatients decreased due to reasons such as postponing surgeries and decreasing hospitalizations because of the risk of infection. Due to the increase in exchange rate and inflation in 2021, problems arose in the purchase of medicines, medical devices and materials [33]. As a result, the number of hospitalized patients and surgeries decreased. In addition, with the decrease in the number of patients in small-scale hospitals, their incomes decreased and therefore physicians and health personnel began to be unable to receive performance based supplementary payment. Therefore, the work motivation of the health workers decreased and this situation negatively affected the service production capacities of the hospitals. As can be seen from the efficiency scores, while outpatient efficiency was higher in small-scale hospitals, inpatient efficiency was quite low.

Moreover, we can see that being TRH or City Hospital have negative effect on hospital outpatient services production efficiency, but positive effect on inpatient services production efficiency. In Türkiye, 50% of beds in hospitals affiliated to the MoH and 75% of intensive care beds are in TRH, and in 2021 almost 75% of the inpatients and surgical procedures were performed in TRH [74]. Thus, TRH’s higher efficiencies in inpatient services is consistent with previous studies [75–77]. Indeed, city hospitals alleviated the burden of the pandemic with the number of beds and intensive care units. Due to the increasing Covid-19 cases, city hospitals served inpatients as reference hospitals, as their physical infrastructure and status of medical devices were sufficient. Since the high number of inpatients for the treatment of Covid-19 disease, the inpatient efficiency was higher than other public hospitals.

In addition, we found that inpatient services efficiency was negatively affected by being located in crowded cities, while outpatient efficiency was fostering. The MoH hospitals with the highest outpatient efficiency scores were in Southeastern Anatolia and Marmara regions. The hospital efficiency in the Black Sea region remained relatively lower because of the low population density. These findings show a similar trend with previous studies [77, 78]. The high efficiency of total in the Southeastern Anatolia region is due to the fact that the number of private hospitals is few and the socio-economic status of the region is low, so patients prefer public hospitals more. It is also the region with the youngest population and with a high fertility rate. This is a factor that increases the number and efficiency of outpatients rather than inpatients. On the contrary, the high number of private hospitals in the Marmara region has relatively reduced inpatient efficiency. For example, while 51.9% of the total inpatients were in MoH hospitals in 2020, the lowest rate was in Istanbul with 44%. This rate was higher in the Black Sea (60.5%) and eastern and Southeastern Anatolia (56%) regions, where the number of private hospitals is relatively lower [56]. Finally, we can see that exchange rate has significant inefficiency effect neither on outpatient nor inpatient services production. Thus, during the examined period health system has been successfully protected from the vulnerability of the Turkish currency. In Türkiye, the MoH set the fixed exchange rate periodically for the imported drugs, such as in 2021, even though the realized average rate was 10.47 €/TRY, the set rate was 4.58€/TRY [79]. This policy was successful during the 2015–2021 period, however as the gap between the realized and the fixed value widens, problem of drug shortages in domestic market is taken place currently; thus, medium and long run sustainability of fixed exchange rate should be carefully investigated in future studies.

This study was conducted under some limitations. First, the study was limited to only measuring the efficiency of MoH public hospitals during 2015–2021. Therefore, future studies should be conducted to measure the efficiency of private and university hospitals. In addition, the validated simulation model can be run to prove the impact of Covid-19 on the clinical and financial situation of hospitals. As our study is the first examining impact of the pandemic on Turkish public hospitals efficiencies, we believe our findings will be benchmark and provide important feedback to policymakers and the researchers. Especially after the Covid-19 pandemic, hospital efficiency has become a greater focus of policymakers and researchers both nationally and internationally. The use of evidence-based recommendation in this study can guide policymakers in improving their response to the pandemic and help researchers develop new insights in the context of the pandemic.

## Data Availability

The authors used the Turkish public hospitals’ data for the years 2015–2021 with official permission from the Ministry of Health of Türkiye that prohibits the sharing of the data with the third parties. The public hospitals data cannot be copied or released to any other person or organization.

## Footnotes

1 The *CMI* calculation is included in the Ministry of Health Diagnostic Groups Information Guide [59].

2 The efficiency scores for the anonymized public hospitals is available and can be given upon request to corresponding author.

3 In Türkiye the regions from most to least crowded are: Marmara, Central Anatolia, Aegean, Mediterranean, Southeast Anatolia, Black Sea, and East Anatolia.
